# Multitrophic diversity in a biodiverse forest is highly nonlinear across spatial scales

**DOI:** 10.1038/ncomms10169

**Published:** 2015-12-10

**Authors:** Andreas Schuldt, Tesfaye Wubet, François Buscot, Michael Staab, Thorsten Assmann, Martin Böhnke-Kammerlander, Sabine Both, Alexandra Erfmeier, Alexandra-Maria Klein, Keping Ma, Katherina Pietsch, Sabrina Schultze, Christian Wirth, Jiayong Zhang, Pascale Zumstein, Helge Bruelheide

**Affiliations:** 1 Institute of Ecology, Leuphana University Lüneburg, Scharnhorst Straße 1, D-21335 Lüneburg, Germany; 2 Department of Soil Ecology, UFZ-Helmholtz Centre for Environmental Research, Theodor-Lieser-Straße 4, D-06120 Halle (Saale), Germany; 3 German Centre for Integrative Biodiversity Research (iDiv) Halle-Jena-Leipzig, Deutscher Platz 5e, D-04103 Leipzig, Germany; 4 Institute of Earth and Environmental Sciences, University of Freiburg, Tennenbacher Straße 4, D-79106 Freiburg, Germany; 5 Institute of Biology/Geobotany and Botanical Garden, University of Halle, Am Kirchtor 1, D-06108 Halle, Germany; 6 Institute of Biological and Environmental Sciences, University of Aberdeen, St Machar Drive 23, AB24 3UU Aberdeen, UK; 7 Institute for Ecosystem Research, University of Kiel, Olshausenstrasse 75, 24118 Kiel, Germany; 8 Institute of Botany, Chinese Academy of Sciences, Beijing 100093, China; 9 Systematic Botany and Functional Biodiversity, University of Leipzig, Johannisallee 21-23, D-04103 Leipzig, Germany; 10 Institute of Ecology, Zhejiang Normal University, Yinbing Road 688, Jinhua 321004, China

## Abstract

Subtropical and tropical forests are biodiversity hotspots, and untangling the spatial scaling of their diversity is fundamental for understanding global species richness and conserving biodiversity essential to human well-being. However, scale-dependent diversity distributions among coexisting taxa remain poorly understood for heterogeneous environments in biodiverse regions. We show that diversity relations among 43 taxa—including plants, arthropods and microorganisms—in a mountainous subtropical forest are highly nonlinear across spatial scales. Taxon-specific differences in β-diversity cause under- or overestimation of overall diversity by up to 50% when using surrogate taxa such as plants. Similar relationships may apply to half of all (sub)tropical forests—including major biodiversity hotspots—where high environmental heterogeneity causes high biodiversity and species turnover. Our study highlights that our general understanding of biodiversity patterns has to be improved—and that much larger areas will be required than in better-studied lowland forests—to reliably estimate biodiversity distributions and devise conservation strategies for the world's biodiverse regions.

## Abstract (web summary)

Measurements of biodiversity may differ according to the extent of area sampled, although how this changes across taxa is not well understood. Here, Schuldt *et al*. find that the diversity of plants, arthropods and microorganisms in a heterogeneous subtropical forest is highly nonlinear across spatial scales.

## Graphical abstract

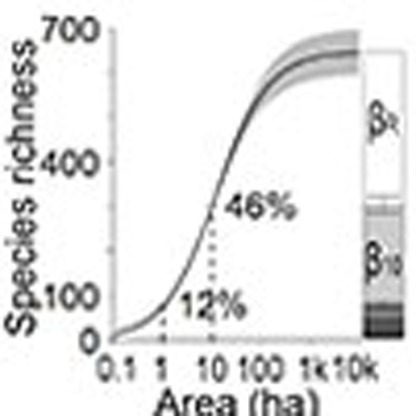

Untangling the scale-dependency of α- and β-diversity among coexisting taxa is essential to understand the structuring of ecological systems, to estimate regional and global species richness, and to inform policy options on conservation^1^,^2^,^3^,^4^,^5^,^6^. However, how exactly megadiverse groups such as arthropods and microorganisms scale in relation to more frequently assessed taxa, such as plants, is a matter of ongoing debate. This particularly applies when extrapolating assessments to landscape and regional scales in the most species-rich terrestrial regions of the world, subtropical and tropical forests^2^,^7^.

Most studies on scale-dependent biodiversity patterns in species-rich forests have focused on single taxa^8^,^9^,^10^,^11^,^12^,^13^. Those studies that have considered multiple taxa have analysed various—including non-forest—habitat types or restricted the spatial analyses to pairwise plot comparisons^14^,^15^,^16^,^17^. Despite their functional importance, microorganisms have so far been ignored in such studies. A recent study in a lowland neotropical rainforest, however, showed similarities in species turnover (β-diversity) for a wide range of arthropod taxa^7^. By extrapolating local plot species inventories, that study showed that areas as small as 1 ha can harbour almost two-thirds of the landscape-scale species richness. Moreover, the species richness of arthropods across all trophic levels was surprisingly well predicted by that of woody plants, and this strong relationship was independent of the geographic scale considered^7^. However, whether these patterns can be extrapolated to species-rich forest types in more heterogeneous environments, and to other species-rich taxa such as microorganisms, is questionable. Many highly diverse forests, and many of the world's biodiversity hotspots^18^, are located in mountainous landscapes with heterogeneous topography, which results in a higher β-diversity of many taxa than in more homogeneous lowland forests^9^,^19^. This may have consequences for the design and costs of biodiversity research and conservation, and for species richness estimates at larger spatial scales^19^,^20^.

We conducted a comprehensive assessment of the species richness, turnover, and cross-taxon diversity congruence of plants, arthropods and, for the first time, soil microorganisms from the local plot to landscape scales in a highly diverse, and topographically and environmentally heterogeneous, subtropical forest. We used multi-method species censuses of above- and below-ground organisms (woody and herbaceous plants; 10 arthropod taxa comprising herbivores, detritivores, predators and parasitoids; 12 groups of soil fungi and 19 groups of bacteria) and modelled species richness and area relationships (Methods). The data were obtained from 27 study plots that reflect the environmental heterogeneity typically found in the 8,000-ha mountainous study site, a national forest reserve in South-East China. Our analysis shows that cross-taxon diversity relationships are highly nonlinear across spatial scales, with far-reaching consequences for our understanding of regional and global biodiversity patterns and their conservation.

## Results

### Sampling completeness

Altogether, we identified 1,008 (morpho)species of arthropods and plants with a total of 77,718 individuals, and 6,223 operational taxonomic units (OTUs) of microorganisms. Species–area relationships for all taxa were best modelled by asymptotic functions. Sample coverage, a measure of sample completeness, approached values >90% relatively fast with increasing plot number, and was on average 97% across all taxa in 27 plots (with the exception of lepidopterans; Supplementary Fig. 1).

### Spatial scaling of species richness patterns

**Figure 1:**
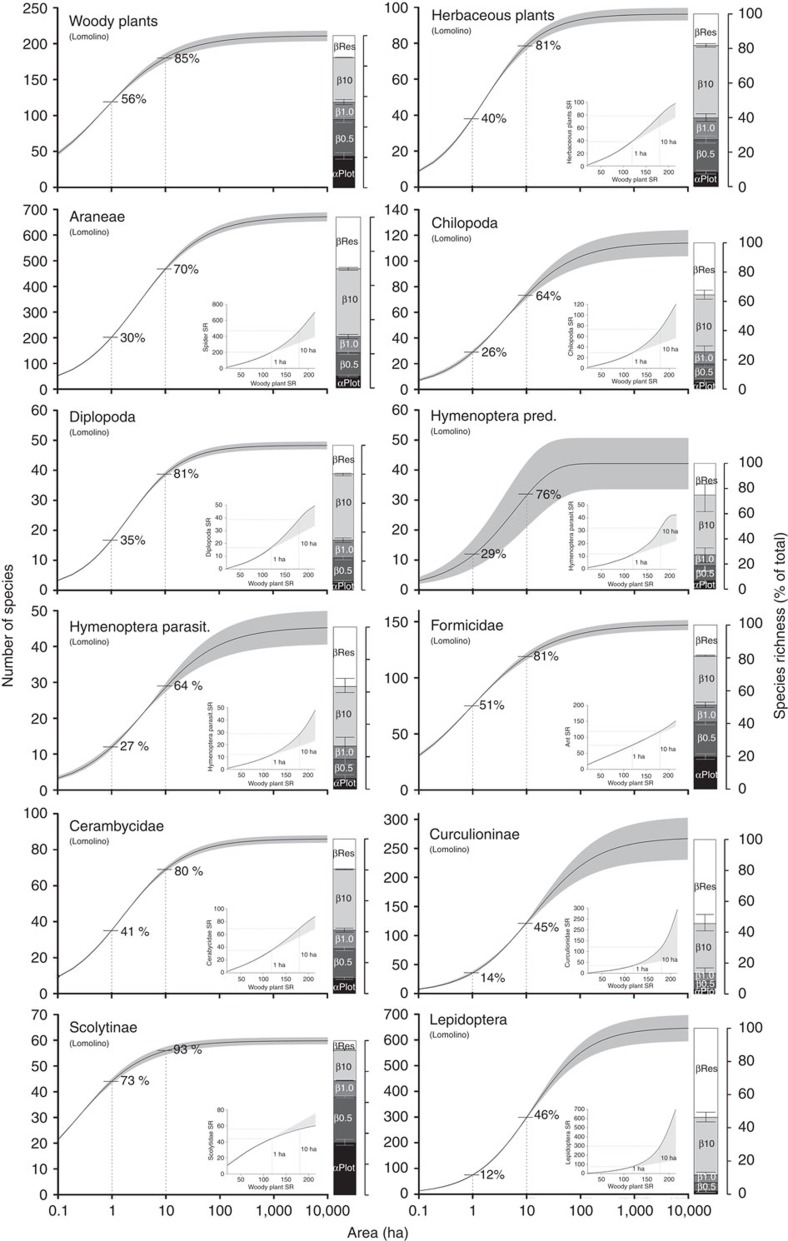
Species–area relationships, turnover and relationships with woody plant species richness for herbaceous plants and arthropods in the Gutianshan National Nature Reserve. Shaded areas in the species–area curves show 95% confidence bands, percentage values are fractions of the total estimated species richness in 1 and 10 ha of forest. Stacked barplots show the average number of species per study plot (α_Plot_; *n*=27) and the relative species turnover (β) at scales of 0.5, 1, 10 ha, and the whole reserve (±95% confidence intervals). Insets below the curves show species-richness relationships between woody plants and herbs or arthropods, based on the species–area models. Shaded areas in the inset show the deviation between the estimated nonlinear relationships across the whole reserve and a linear relationship based on the species richness data of ≤1 ha.

**Figure 2:**
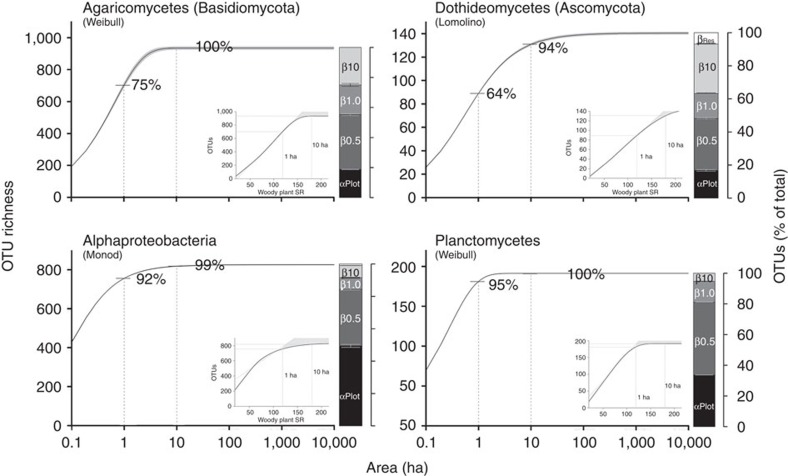
Species–area relationships, turnover and relationships with woody plant species richness for selected taxa of fungi and bacteria. The taxa shown reflect the variation in spatial turnover patterns exhibited by the 12 fungal (top) and 19 bacterial (bottom) taxa analysed. Shaded areas in the species–area curves show 95% confidence bands, percentage values are fractions of the total estimated species richness in 1 and 10 ha of forest. Stacked barplots show the average number of species per study plot (α_Plot_; *n*=27) and the relative species turnover (β) at scales of 0.5, 1, 10 ha, and the whole reserve (±95% confidence intervals). Insets below the curves show species-richness relationships between woody plants and microorganisms, based on the species–area models. Shaded areas in the inset show the deviation between the estimated nonlinear relationships across the whole reserve and a linear relationship based on the species richness data of ≤1 ha.

On average, 1 and 10 ha of the subtropical forest at our study site can be expected to capture 38% and 76%, respectively, of the overall estimated species richness for the 10 arthropod taxa, 71% and 97% for the 12 fungal taxa and 93% and 100% of the 19 bacterial taxa (Figs 1 and 2, Supplementary Figs 2 and 3 and Supplementary Table 1). Halving the mean distance between plots by limiting the analysis to a reduced set of spatially closer plots only yielded a slight increase in these proportions (by an average of 0.9% for all taxa and 5.1% for arthropods; Supplementary Table 1). However, at any given spatial scale, the fractions of overall estimated species richness, and thus the degree of species turnover, differed strongly among taxa (Fig. 1). Most arthropod taxa showed highest turnover rates between scales of 1 and 10 ha (Fig. 1 barplots). However, turnover in weevils and lepidopterans was highest at scales >10 ha, and in bark beetles and all microorganisms at scales ≤1 ha (Fig. 1). Differences in turnover patterns among fungi and among bacteria were much less pronounced and much more similar than among arthropods (Fig. 2 and Supplementary Figs 2 and 3). However, two different turnover patterns emerged for both fungi and bacteria: taxa with very little turnover at scales >1 ha (‘Fungi 1': for example, Archaeorhizomycetes, Leotiomycetes, Agaricomycetes) and taxa where large-scale turnover was slightly more noticeable (‘Fungi 2': for example, Dothideomycetes, Eurotiomycetes, Tremellomycetes). Likewise, for some bacterial taxa, turnover was particularly high at the plot scale (‘Bacteria 1': for example, Acidobacteria, Alphaproteobacteria, Cyanobacteria), whereas for others it was highest at the scale of 0.5 ha (‘Bacteria 2': for example, Bacteroidetes, Deltaproteobacteria, Planctomycetes; Fig. 2 and Supplementary Figs 2 and 3).

### Cross-taxon congruence

**Figure 3:**
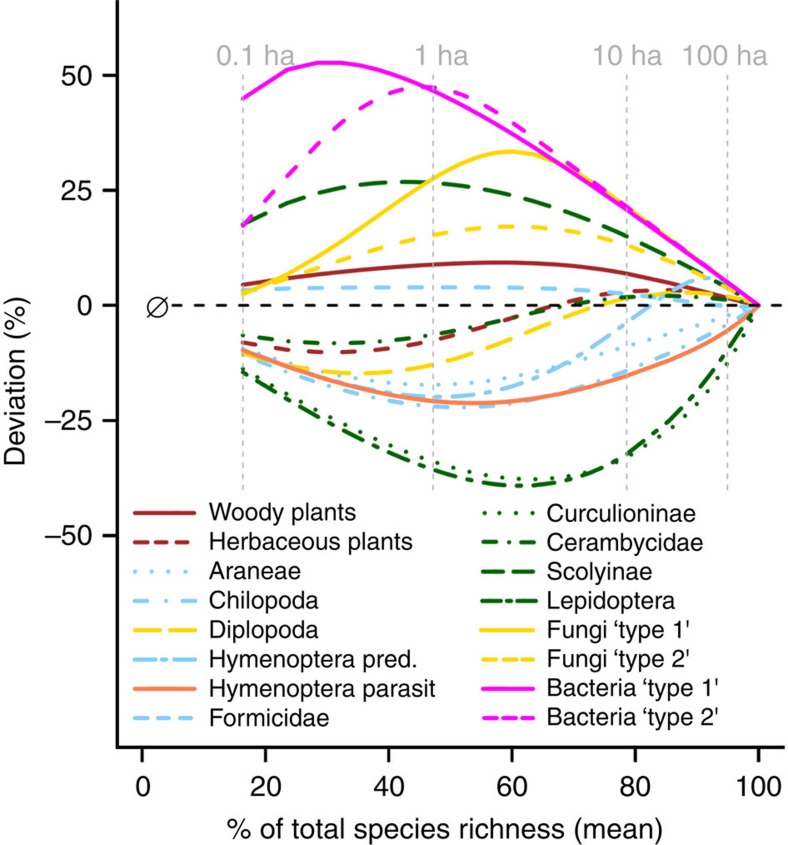
Deviation of relative species richness patterns among the focal taxa. The plot shows the extent to which the species richness of a given taxon over- or underestimates the species richness of the other taxa relative to the overall species richness of each taxon. For example, the +50% deviation for bacteria ‘type 2' at the scale of 1 ha indicates that the species richness of bacteria would underestimate overall relative species richness patterns by 50%. For the 12 fungal and 19 bacterial taxa, only 4 taxa representing the most common types of turnover patterns are shown (Fungi ‘type 1': Agaricomycetes; Fungi ‘type 2': Dothideomycetes; Bacteria ‘type 1': Alphaproteobacteria; Bacteria ‘type 2': Planctomycetes).

The differences in species turnover also affected the congruence of species richness patterns among taxa. For most taxa, linear species richness predictions of a given taxon based on woody plant species richness showed greater deviation with increasing spatial scale (Figs 1 and 2 insets). These nonlinear relationships resulted from higher species turnover of arthropods, but lower turnover of fungi and particularly bacteria, at larger scales, as compared with that of woody plants. However, patterns of relative species richness (that is, relative to the overall estimated species richness) across scales also differed among arthropods and microorganisms, and no taxon qualified unambiguously as a surrogate for the species richness of all other taxa (Fig. 3).

## Discussion

Our study indicates that environmentally heterogeneous forests are characterized by a substantial turnover in the species richness of many taxa. Capturing a percentage of overall biodiversity similar to that obtained in 1 ha of lowland tropical forests^7^ would require much larger areas of up to 10 ha at our study site. Similar relationships may apply to a broad range of forest ecosystems in the subtropics and tropics, where high environmental heterogeneity promotes high overall biodiversity and causes higher rates of spatial species turnover than in lowland forests^9^,^19^. This also includes major biodiversity hotspots^21^, emphasizing that we still lack a general understanding of diversity relationships in those regions of the world that are assumed to be the most diverse^22^. Our findings underline that a better ecological understanding of scale-dependent biodiversity relationships in heterogeneous landscapes is needed if we aim to predict larger-scale biodiversity distributions in the most biodiverse regions on the Earth. This also applies to estimates of regional and global species numbers, which, so far, have been based largely on relatively homogeneous landscapes, such as lowland tropical forests^2^,^7^,^19^. Focusing on homogeneous lowland forests ignores the fact that 47% of tropical and subtropical moist broadleaf forests are located in mountainous regions that are environmentally more heterogeneous (with a mean elevation of 997 m±689 s.d.) than typical lowland forests (making up 53% of the forest area, at a mean elevation of 220 m±189 s.d.; see Methods for details). Moreover, our study suggests that the logistic efforts and costs required for the implementation of reliable biodiversity assessments will inevitably increase. The same applies to conservation planning and the estimation of minimum areas required for the protection of the overall biodiversity of a larger region^20^. In this respect, it might also be important to consider that the observed differences in turnover among taxa might affect the way the diversity and functions mediated by a given taxon are influenced by local disturbances, such as logging^23^.

The nonlinear diversity relationships and scale-dependent changes in cross-taxon congruence indicate that it is not advisable to use single taxa as potential surrogates of overall biodiversity^14^,^24^. Nevertheless, our results indicate that there might be distinct groups of taxa that show remarkable similarities in the scale-dependent increase of relative species richness (that is, herbs, diplopods and longhorn beetles; woody plants and ants; weevils and lepidopterans; spiders, centipedes and parasitic wasps; Fig. 3). This might allow for the identification of a complementary set of indicator taxa that can be used to infer diversity patterns for a wider range of organisms^25^,^26^. Such an approach would help to better estimate and monitor overall biodiversity patterns and thus guide management decisions in poorly studied, species-rich regions. However, its effectiveness and transferability to other systems requires further investigation. Contrasting cross-taxon relationships have been reported from lowland tropical forests^7^,^27^, with even non-herbivorous arthropods showing a strong linear and apparently scale-independent increase with plant species richness^7^. This might indicate that in lowland forests with a more homogenous abiotic environment, plant species richness is the most important driver of arthropod species richness^7^,^27^.

In contrast, environmental heterogeneity can affect interactions among trophic levels^28^ and decrease the impact of plant species richness relative to other environmental drivers^19^. Our results suggest that differences in the environmental conditions throughout our study site (and/or dispersal-based processes^13^) affect arthropods more strongly than woody plants. Even for herbivorous insects, patterns of species richness do not necessarily match those of their host plants in environmentally heterogeneous forests. Other factors, such as plant biomass and topographically mediated environmental conditions, can be crucial in determining herbivore species richness^19^. A direct analysis of the ultimate drivers for the wide range of taxa (43) is beyond the scope of our study, as we address key aspects of diversity scaling with regard to biodiversity estimation and conservation, which presently lack a proper integration of highly diverse and environmentally heterogeneous ecosystems. However, environmental conditions in our study system vary across scales in predictable ways and might be indicative of the drivers underlying the observed differences in spatial diversity scaling (Supplementary Fig. 4; with for example, elevation and temperature related to large-scale turnover patterns such as in lepidopterans and weevils, soil N and C concentrations to medium-scale turnover such as in woody plants, and slope and pH with small-scale turnover such as in bark beetles or microorganisms). Understanding the scale dependence of the environmental drivers of the different taxa will be crucial for advanced insights into community assembly processes and the consequences of increasing anthropogenic impact on diversity distributions. Taxon-specific dispersal abilities^13^ and the niche breadth and degree of resource specialization of species^20^,^29^ are likely to play an important role in this respect. For instance, saproxylic bark beetles show very low host specificity^30^, and the diversity of saproxylic beetles can be largely determined by the availability of deadwood^29^. In a protected forest reserve such as the Gutianshan National Nature Reserve (GNNR), these resources are readily available throughout the forest and thus could lead to high levels of local-scale diversity and low spatial turnover of bark beetles. In contrast, many herbivore arthropods show a higher niche specialization^31^ and even generalist predators such as spiders, while often less specific in their food requirements, have been shown to exhibit pronounced and fine-scaled adaptations to structural and microclimatic conditions^32^ that can result in high spatial species turnover. For soil microorganisms, the lower spatial turnover of bacteria and, to a lesser extent, soil fungi as compared with that of plants is concordant with global distribution patterns and can be partly attributed to their high dispersal capacity^33^. Moreover, soil microorganisms might respond to environmental heterogeneity on much smaller scales than aboveground macroorganisms^34^. This likely leads to a high turnover at scales much smaller than those considered in our study, particularly for bacteria, but does not affect our findings for the spatial scales considered in our analysis. Although more fine-scaled sampling within plots would thus probably reveal additional insights at the sub-plot level, our sampling approach for microorganisms, with four replicate composite samples per plot, aggregates microsite variation at the plot level and enables adequate comparison with arthropods and plants at the plot level and beyond. However, the indication of two different spatial turnover patterns for fungal and bacterial groups that comprise taxa with differing functional or ecological characteristics (for example, Agaricomycetes and Alphaproteobacteria) suggests the need for further detailed analysis of small-scale distribution patterns at the level of more finely resolved functional groups.

Although our study covers all strata from the soil to the canopy, most taxa were sampled in the lower forest strata. A complete species census for the estimation of the total species richness at our study site is therefore not available and was not our aim. Considering that the species richness of lower forest strata can equal that of the canopy in species-rich forests^35^,^36^, the relationships we report may nevertheless be relevant for total biodiversity patterns. Moreover, results did not differ qualitatively between taxa with an expected high species richness in the canopy (for example, spiders, weevils, longhorn beetles) and taxa largely restricted to lower strata, for which a large proportion of the total diversity has likely already been sampled (for example, diplopods, herbs; Fig. 1). We note that our study has a strong focus on functional groups (for example, taxa with specific feeding ecologies in arthropods), which may differ in their Linnean rank. These taxonomic ranks can suffer from inconsistencies when it comes to comparisons among different taxa^37^, and our study thus adopts an ecological rather than strictly taxonomic perspective (but note that our data on microorganisms, while analysed at the OTU level, represent a comparatively coarse distinction of taxonomic and ecological groups). As we analysed the relative scaling of species richness patterns (rather than comparing absolute richness values or analysing community composition patterns), our results are unlikely to be strongly affected by the potential variation in abundance patterns of individual species in a community between different sampling years.

The nonlinear relationships we observed complicate attempts for regions of high environmental heterogeneity to reliably predict diversity across multiple trophic levels based on plant inventories. This indicates that upscaling species richness relationships from smaller (for example, data from 1 ha) to larger scales, and vice versa, downscaling from regional and landscape scale assessments to local-scale relationships, can be problematic^24^,^38^ and emphasizes the need for more intensive studies of biodiversity patterns in the world's most biodiverse regions.

## Methods

### Study site and study design

The GNNR is located in Zhejiang province, South-East China (29°14′ N; 118°07′ E). It covers ∼8,000 ha of evergreen mixed broadleaved forest on sloping terrain (300–1,260 m above sea level), with *Schima superba* Gardn. et Champ. and *Castanopsis eyrei* (Champ. ex Benth.) Tutch. as the dominant tree species. The climate is subtropical, with a mean annual temperature of 15.3 °C and a mean annual precipitation of ca 2,000 mm (ref. 39).

In 2008, 27 study plots (30 × 30 m^2^) were established in the reserve using a stratified sampling design to capture the range of woody plant species richness (25–68 species per plot) and successional age (from <20 to >80 years since the last logging events) typically encountered in the reserve. The study plots were randomly spread across the accessible parts of the GNNR (see ref. 40 for details). Owing to the topography of the study site, environmental heterogeneity among the study plots is much more pronounced than in similarly diverse lowland forests (see Supplementary Table 2 and Supplementary Fig. 4 for details).

### Species sampling

Our study focused on the species richness patterns of 43 taxa that cover a large range of trophic levels and that, in many cases, represent specific functional groups (for example, similar feeding ecologies in arthropods)—a selection criterion commonly used in biodiversity studies^7^,^9^,^14^,^16^: woody plants, herbaceous plants, spiders (Arachnida: Araneae), centipedes (Chilopoda), millipedes (Diplopoda), cavity-nesting solitary wasps (Hymenoptera: Pompilidae, Sphecidae, Vespidae), parasitic wasps (Hymenoptera parasitica: Braconidae, Chrysididae, Eurytomidae, Ichneumonidae, Leucospidae, Megachilidae, Mutillidae, Pompilidae, Trigonalyidae), ants (Hymenoptera: Formicidae), longhorn beetles (Coleoptera: Cerambycidae), weevils (Coleoptera: Curculioninae), bark beetles (Coleoptera: Scolytinae), moths and butterflies (Lepidoptera), as well as 12 groups of fungi (at the class level; Ascomycota: Arachaeorhizimycetes, Dothideomycetes, Eurotiomycetes, Leotiomycetes, Orbiliomycetes, Sordariomycetes, Ascomycota incertae sedis; Basidiomycota: Agaricomycetes, Tremellomycetes, Wallemiomycetes; Glomeromycetes, Zygomycota) and 19 groups of bacteria (at the phylum and for Proteobacteria at the subphylum level; Acidobacteria, Actinobacteria, Alphaproteobacteria, Armatimonadetes, Bacteroidetes, Betaproteobacteria, Chlorobi, Chloroflexi, Cyanobacteria, Deltaproteobacteria, Elusimicrobia, Firmicutes, Gammaproteobacteria, Gemmatimonadetes, Planctomycetes, TM6, Verrucomicrobia, WCHB1-60, WD272). Permission for sampling was granted by the Administration Bureau of the Gutianshan National Nature Reserve, Zhejiang, China.

The woody plant species richness of each plot was assessed by means of a complete inventory of all tree and shrub individuals >1 m height, conducted during plot establishment in 2008 (ref. 40). At the same time, the species richness of herbaceous plants was assessed in the central 10 × 10 m^2^ of each plot, in the form of abundance and cover estimates of all herb layer species (<1 m height; see ref. 41).

Arthropods were sampled with a range of different techniques during the main growing seasons of the years 2008–2012. Epigeic spiders, chilopods, diplopods, epigeic ants and weevils were sampled with pitfall traps from March to September 2009 (4 traps per plot and a total of 15,704 trap days; see ref. 42 for details on the trapping design). Lepidopteran larvae and arboreal spiders and ants were sampled by beating from understory trees and shrubs in 2011 and 2012 (25 plant individuals per plot on three sampling dates, that is, a total of 2,025 tree and shrub individuals; see ref. 43 for details). Cavity-nesting predatory wasps and the associated parasitic wasps were sampled with reed-filled trap nests from September 2011 to October 2012 (2 traps per plot and a total of 21,720 trap days; see ref. 44 for details). Longhorn beetles, bark beetles and canopy ants were sampled by means of flight interception traps from May to August 2010 (4 traps consisting of two crossed Plexiglas panels (50 × 30 cm^2^), a funnel ending in a replaceable PE-bottle filled with trapping solution and attached below the panels, with a total of 13,608 trap days). In addition, ants were sampled by placing pairs of standardized protein and carbohydrate baits on the ground and at breast height in the middle of each 10 × 10 m^2^ subplot in May 2012 (that is, 36 baits per plot; see ref. 45 for details). All arthropods were identified to morphospecies (in part based on their genitalia, for example, spiders) or, where possible, species (for example, many Hymenoptera). Taxon-specific data from the different methods were pooled per plot. Together with spreading replicate sampling points for the individual methods within the study plots, this ensured that a large proportion of the overall species richness per taxon in each plot was included in the analyses (see also ref. 7 for details).

Soil samples to determine microorganism diversity (fungi and bacteria) were collected from the upper 0–10 cm of soil (eight samples from different locations within each plot, pooled to four composite samples per plot to obtain composite samples that aggregate microsite variation at the plot level for an adequate comparison with plot-level arthropod and plant data) in September 2012. Soil cores were sieved, cool transported to the field lab and freeze dried for further molecular analysis. Microbial DNA was extracted from 1 g of each of the composite freeze-dried soil sample using the MoBio soil DNA extraction kit. The presence and quantity of genomic DNA were checked using a NanoDrop ND-1000 spectrophotometer (Thermo Fisher Scientific). DNA extracts were then stored at −20 °C for further analysis. Bacterial and fungal amplicon libraries were amplified for pyrosequencing using custom fusion primers. We used the primer pairs BAC 341F (5′-CCTACGGGAGGCAGCAG-3′) and BAC 907R (5′-CCGTCAATTCMTTTGAGTTT-3′) to amplify the V3–V5 region of the bacterial 16S rRNA gene^46^,^47^. We used the primer pairs ITS1F^48^ (5′-CTTGGTCATTTAGAGGAAGTAA-3′) and ITS4 (ref. 49; 5′-TCCTCCGCTTATTGATATGC-3′) to amplify the fungal internal transcribed spacer (ITS) rRNA region. The custom primers were constructed with the barcodes and sequencing primers attached at the BAC 907R and ITS4 primers for unidirectional sequencing (see details for the PCR conditions in refs 50, 51, 52). PCR products were checked by separation on a 1.5% agarose gel electrophoresis and purified by gel extraction using the QIAquick Gel Extraction Kit (QIAGEN). The purified DNA was quantified using a fluorescence spectrophotometer (Cary Eclipse, Agilent Technologies). An equimolar mixture of each library was subjected to unidirectional pyrosequencing from the 907R and ITS4 ends of the amplicons, using a 454 Titanium amplicon sequencing kit and a Genome Sequencer FLX+ 454 System (454 Life Sciences/Roche Applied Biosystems).

Fungal and bacterial communities were analysed by pyrotag amplicon sequencing of the fungal ITS^53^ and the V3–V5 region of the bacterial 16S rRNA genes^50^. Sequence data sets were further quality filtered, normalized to enable an unbiased comparison among plots to 10,000 fungal ITS and 20,000 bacterial 16S rDNA reads per plot using MOTHUR^54^. Sequences were clustered into species-level OTUs using CD-HIT-EST at 97% pairwise similarity threshold^50^. Bacterial 16S OTU representative sequences were assigned taxonomy against the Silva SSU reference database, whereas fungal ITS OTU representative sequences were classified against the UNITE database^33^. Non-target taxa OTUs were removed from both data sets. Procrustes analysis (*protest* function of the R package vegan^55^) showed that neither fungal (*r*=0.9944; *P*<0.001) nor bacterial community composition (*r*=0.9988; *P*<0.001, suggesting nearly identical ordinations) were significantly affected by the presence or absence of rare OTUs. Hence, rare OTUs (singletons, doubletons and tripletons), which have a high probability of originating from artificial sequencing errors^56^, were removed from the data set and the abundant OTU's (OTU's with>3 reads per sample) were used for further statistical analysis. Rarefaction analysis indicated that, on average, 74% (±0.5 s.e.) and 75% (±0.7 s.e.), respectively, of the expected number of OTUs per plot for bacteria and fungi were sampled, meaning that both taxa were well-sampled and that the sampling depth was comparable among both taxa with this procedure (Supplementary Fig. 5).

### Statistical analyses

The total number of species expected for each taxon in the GNNR and the increase in species richness with area were modelled with nonlinear species–area models. Unlike other species richness estimators, species–area models allow for explicit estimates of species richness for a specified area size. Our study design provided 27 non-contiguous plots (‘Type IIIB' sampling scheme after ref. 57) that allow for species richness estimates for the cumulative area of an increasing number of equal-sized sampling plots. Such a non-contiguous and non-spatially explicit design is recommended when the aim is to estimate species richness relationships for larger areas, especially in environmentally heterogeneous areas^57^. We calculated the cumulative average number of species per cumulative area of the study plots (from one up to all 27 plots, with randomized ordering of the plots) for each of the 43 taxa. We used the incidence-based function ‘poolaccum' in the R-package *vegan*^55^ and 999 permutations of sampling order.

Species–area relationships with a non-contiguous design can be fitted by a range of different nonlinear functional forms and frequently show better fit with asymptotic functions (such as the Lomolino or Weibull functions) than with the ‘classical' form of a non-asymptotic power function^57^,^58^. We therefore followed the approach of ref. 58 (see also ref. 7) and used an information theoretic-based model selection framework that evaluated the fit of eight different asymptotical and non-asymptotical functions to the species–area data of each of our 43 focal taxa (power, exponential, negative exponential, Monod, rational, logistic, Lomolino and cumulative Weibull function; details and model formulae are specified in ref. 59). We used the R package *mmSAR*^59^ to select the model with the lowest AICc (Akaike Information Criterion corrected for small sample sizes) for each taxon. Models that indicated non-normality or heteroscedasticity of the residuals with the model validation procedure implemented in *mmSAR* were excluded in the model selection procedure^59^. Based on the best-fitting model, species richness data were then extrapolated to the 8,000 ha of the GNNR. In our analyses, we paid special attention to the 1 and 10 ha scales. The 1 ha scale was suggested in a previous analysis in tropical forests to capture almost two-thirds of the overall arthropod species richness^7^. We considered the 10 ha scale to be particularly relevant for our analyses as our study site is environmentally much more heterogeneous than many lowland tropical forest sites. Although model uncertainty may arise if two or more models have similar AICc values, this was not the case in our study and a single best-fitting function (with a ΔAICc>2) was identified in all of our analyses.

As the distance among plots might affect the extrapolation results^60^, we re-ran the analyses with a reduced set of 17 of the 27 plots located in the core area of the reserve (approximately halving the average distance between plots from 3.4 to 1.8 km). This enabled us to check to what extent the relative increase in species richness with area of our 43 focal taxa was potentially influenced by the spatial arrangement of the plots.

As a component of β-diversity, we calculated the effective species turnover across five scales (∼0.1 ha as the mean plot scale, 0.5, 1, 10 and 8,000 ha of the whole reserve) using the additive partitioning method of overall species richness (in our case the estimated species richness for the whole reserve) after ref. 61. Therefore, overall species richness *γ*=α_Plot_+β_Plot_+β_0.5 ha_+β_1 ha_+β_10 ha_+β_Reserve_.

We used two different approaches to analyse the cross-taxon congruence of species richness patterns of the focal taxa. First, plant species richness is commonly assumed to strongly influence the species richness of other organism groups, and woody plants characterize forest ecosystems. We therefore assessed the extent to which the species richness of herbaceous plants, arthropods and microorganisms deviates from an expected linear relationship with woody plant species richness across scales from the local plot scale (∼0.1 ha) to the landscape scale (the 8,000 ha of the whole reserve). For the linear relationship, we extrapolated the relationship predicted by a linear regression between the species richness of woody plants and that of a given taxon at the 1-ha scale to the area of the whole reserve (see insets in Fig. 1 for details), as other studies have suggested a close fit of arthropod species richness with that of woody plants at this scale^7^. Second, allowing for nonlinear relationships among the species richness of the focal taxa, we compared the percent deviation of the relative species richness of each taxon to the mean relative species richness across all taxa (and thus the relative differences among all taxa) from the plot scale to the spatial scales at which ∼100% of the focal taxa's species richness was attained (see Fig. 2 for details).

To estimate sample completeness of the species richness of the focal taxa, we calculated sample coverage according to ref. 62. Sample coverage (equation 4a in ref. 62) indicates the degree of sample completeness by estimating the proportion of the total number of individuals in a sample that belong to the species recorded in this sample. The higher the sample coverage, the less likely it is that an additionally sampled individual will belong to a previously unrecorded species. We calculated sample coverage for all possible combinations (or, as the number of combinations was excessively large for combinations of 6 to 21 plots, for a random draw of 100,000 combinations) of 1, 2, 3,... 27 plots.

Moist broadleaf forest cover was calculated from the percentage cover of planar (<500 m a.s.l.) and montane moist broadleaf forest (>500 m a.s.l.) in the tropics and subtropics, and the mean elevation (±s.d.) of these regions, by overlaying the outline of the ‘Tropical and Subtropical Moist Broadleaf Forests' region provided by ref. 63 with elevation data provided by ref. 64. We calculated the overall area covered by grid cells in planar and montane forest and averaged the elevation data per grid cell for both.

All analyses were conducted in R 3.1.0 (http://www.r-project.org).

## Additional information

**Data deposition statement:** The pyrosequencing data sets of soil bacteria are deposited in the EMBL SRA database under study number PRJEB8980 (http://www.ebi.ac.uk/ena/data/view/PRJEB8980) and likewise the pyrosequencing data set of soil fungi are deposited under the study number PRJEB8979 (http://www.ebi.ac.uk/ena/data/view/PRJEB8979).

**How to cite this article:** Schuldt, A. *et al*. Multitrophic diversity in a biodiverse forest is highly nonlinear across spatial scales. *Nat. Commun.* 6:10169 doi: 10.1038/ncomms10169 (2015).

## Supplementary Material

Supplementary Information Supplementary Figures 1-5, Supplementary Tables 1-2 and Supplementary References.

Supplementary Data 1 Environmental characteristics of the 27 study plots.
