# Supplementary material for: Multitrophic diversity in a biodiverse forest is highly nonlinear across spatial scales
Source: Nat Commun. 2015 Dec 10;6:10169. doi: 10.1038/ncomms10169 (PMC4682160; doi:10.1038/ncomms10169)
Supplement: Supplementary Information — Supplementary Figures 1-5, Supplementary Tables 1-2 and Supplementary References. [file ncomms10169-s1.pdf]

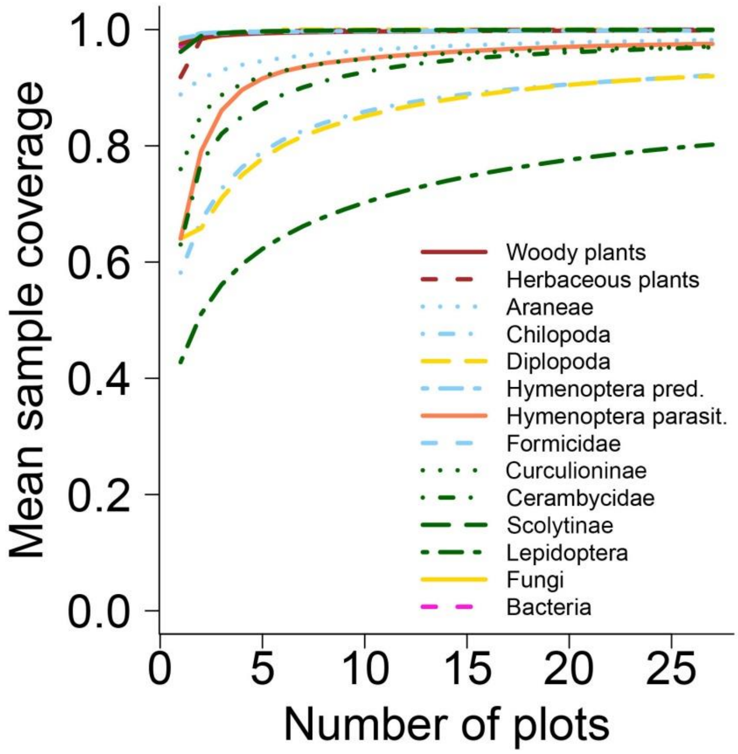

Supplementary Figure 1 | Increase in sample coverage with the cumulative number of study plots for the focal taxa. For clarity, only the overall patterns for fungi and bacteria are shown.

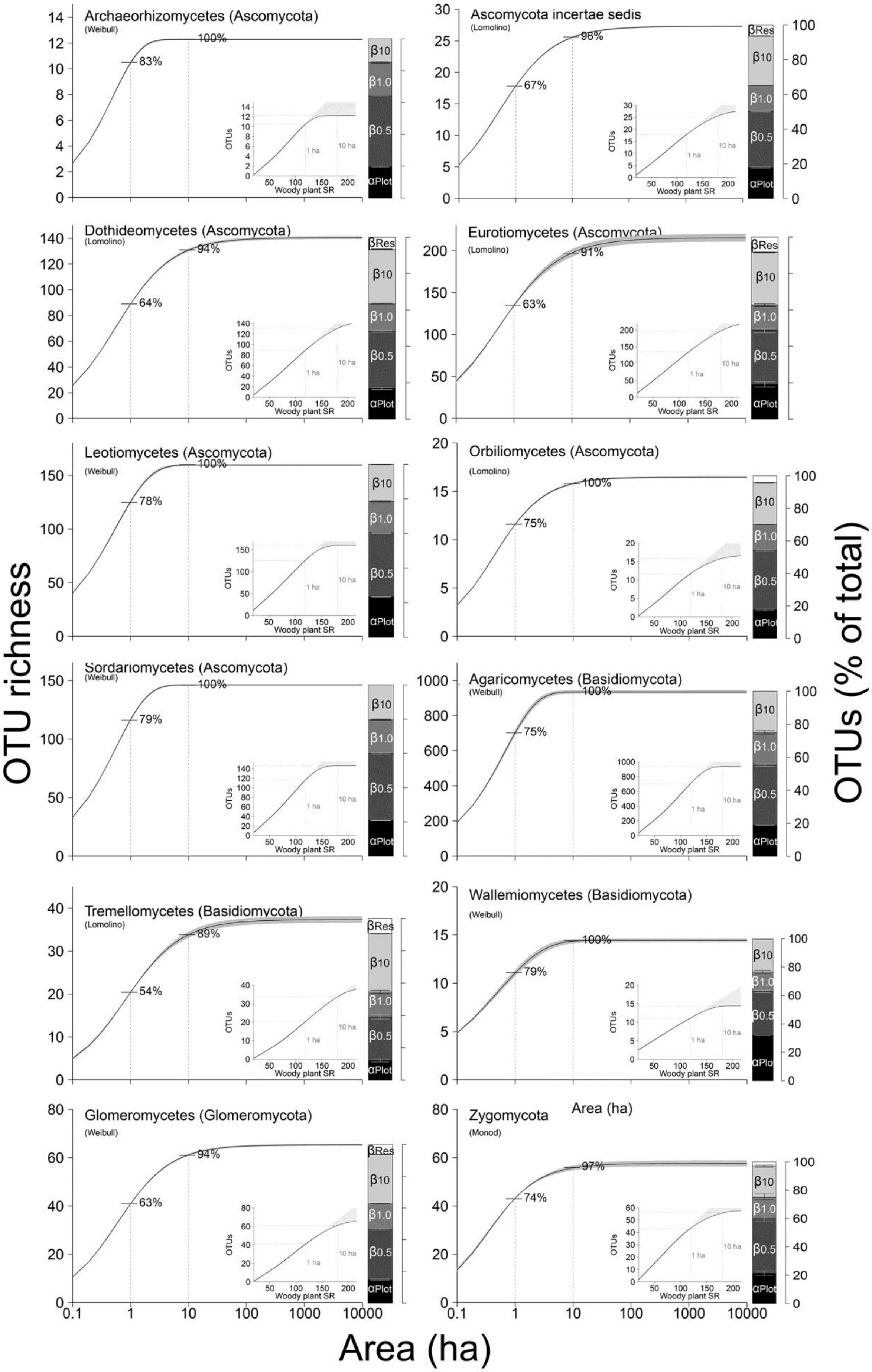

Archaeorhizomycetes (Ascomycota)
(Weibull)
83%
100%
Ascomycota incertae sedis
(Lomolino)
67%
96%
Dothideomycetes (Ascomycota)
(Lomolino)
64%
94%
Eurotiomycetes (Ascomycota)
(Lomolino)
63%
91%
Leotiomycetes (Ascomycota)
(Weibull)
78%
100%
Orbiliomycetes (Ascomycota)
(Lomolino)
75%
100%
Sordariomycetes (Ascomycota)
(Weibull)
79%
100%
Agaricomycetes (Basidiomycota)
(Weibull)
75%
100%
Tremellomycetes (Basidiomycota)
(Lomolino)
54%
89%
Wallemiomycetes (Basidiomycota)
(Weibull)
79%
100%
Glomeromycetes (Glomeromycota)
(Weibull)
63%
94%
Zygomycota
(Monod)
74%
97%
βRes
β10
β1.0
β0.5
αPlot
OTUs
Woody plant SR
1 ha
10 ha
OTU richness
OTUs (% of total)
Area (ha)

Supplementary Figure 2 | Species-area relationships, turnover, and relationships with woody plant species richness for all 12 taxa of fungi. Shaded areas in the species-area curves show 95% confidence bands, percentage values are fractions of the total estimated species richness in 1 and 10 ha of forest. Stacked barplots show the average number of species per study plot (α_{Plot}; n = 27) and the relative species turnover (β) at scales of 0.5 ha, 1 ha, 10 ha, and the whole reserve (± 95% confidence intervals). Insets below the curves show species-richness relationships between woody plants and fungi, based on the species-area models. Shaded areas in the inset show the deviation between the estimated non-linear relationships across the whole reserve and a linear relationship based on the species richness data of ≤ 1 ha.

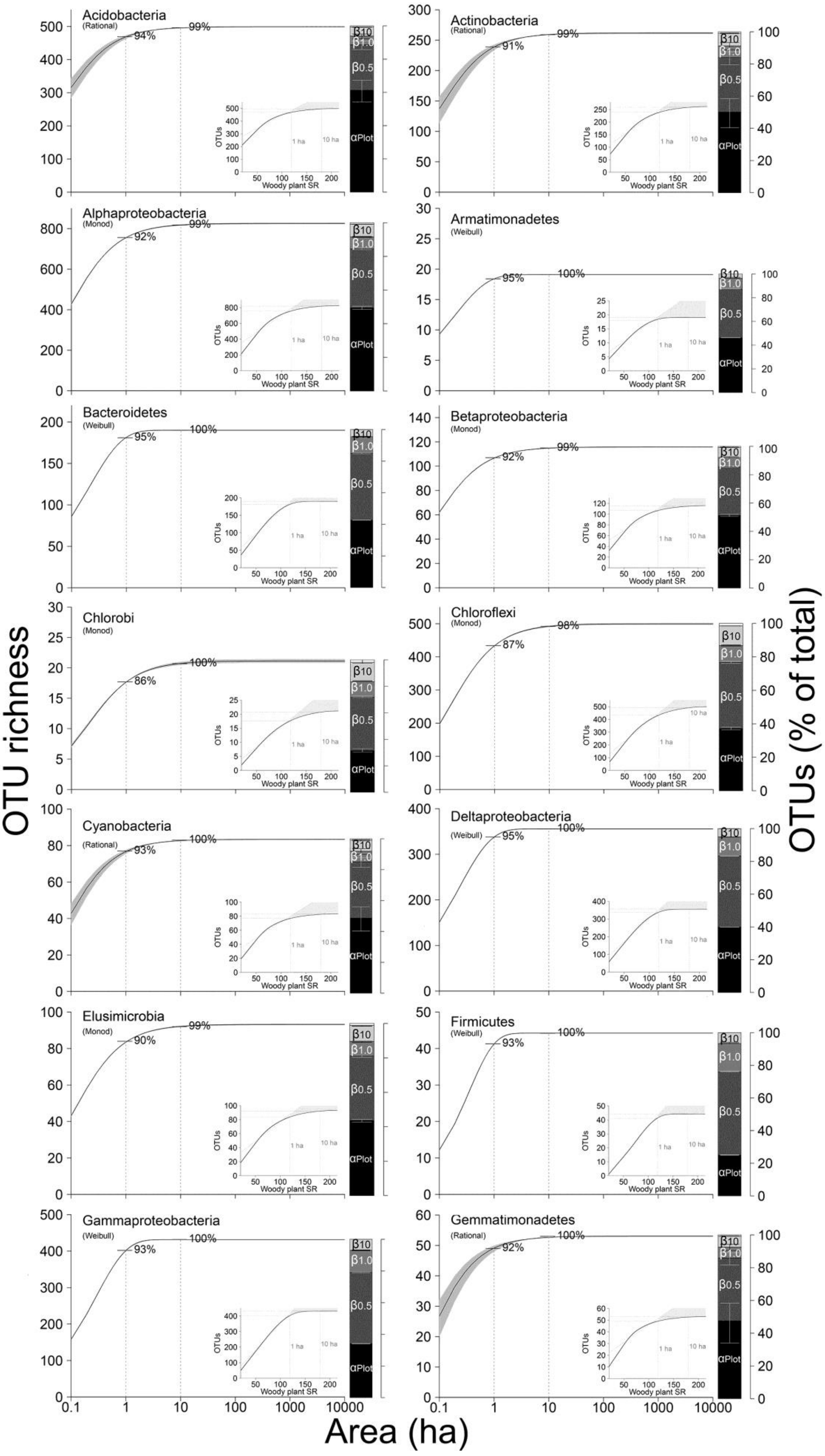

OTU richness
OTUs (% of total)
Area (ha)
Acidobacteria
(Rational)
Actinobacteria
(Rational)
Alphaproteobacteria
(Monod)
Armatimonadetes
(Weibull)
Bacteroidetes
(Weibull)
Betaproteobacteria
(Monod)
Chlorobi
(Monod)
Chloroflexi
(Monod)
Cyanobacteria
(Rational)
Deltaproteobacteria
(Weibull)
Elusimicrobia
(Monod)
Firmicutes
(Weibull)
Gammaproteobacteria
(Weibull)
Gemmatimonadetes
(Rational)
β10
β1.0
β0.5
αPlot
OTUs
Woody plant SR
1 ha
10 ha

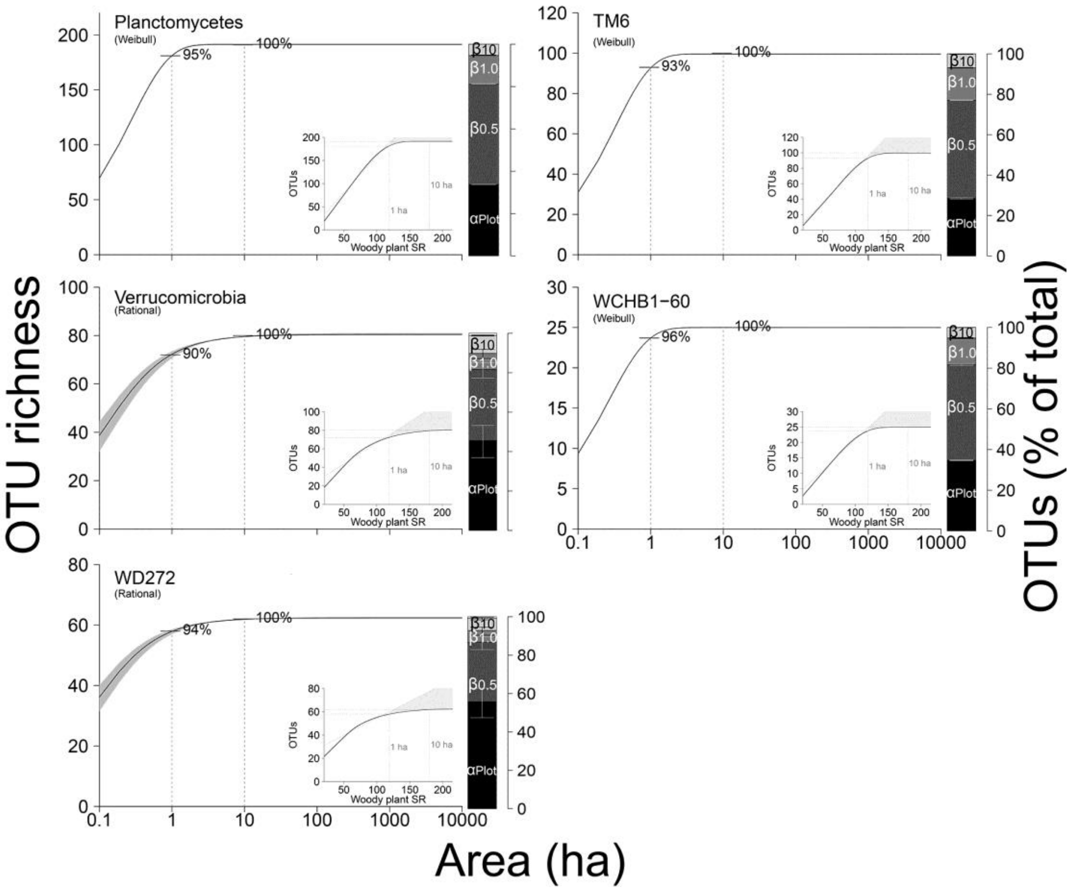

Supplementary Figure 3 | Species-area relationships, turnover, and relationships with woody plant species richness for all 19 taxa of bacteria. Shaded areas in the species-area curves show 95% confidence bands, percentage values are fractions of the total estimated species richness in 1 and 10 ha of forest. Stacked barplots show the average number of species per study plot (α_{Plot}; n = 27) and the relative species turnover (β) at scales of 0.5 ha, 1 ha, 10 ha, and the whole reserve (± 95% confidence intervals). Insets below the curves show species-richness relationships between woody plants and bacteria, based on the species-area models. Shaded areas in the inset show the deviation between the estimated non-linear relationships across the whole reserve and a linear relationship based on the species richness data of ≤ 1 ha.

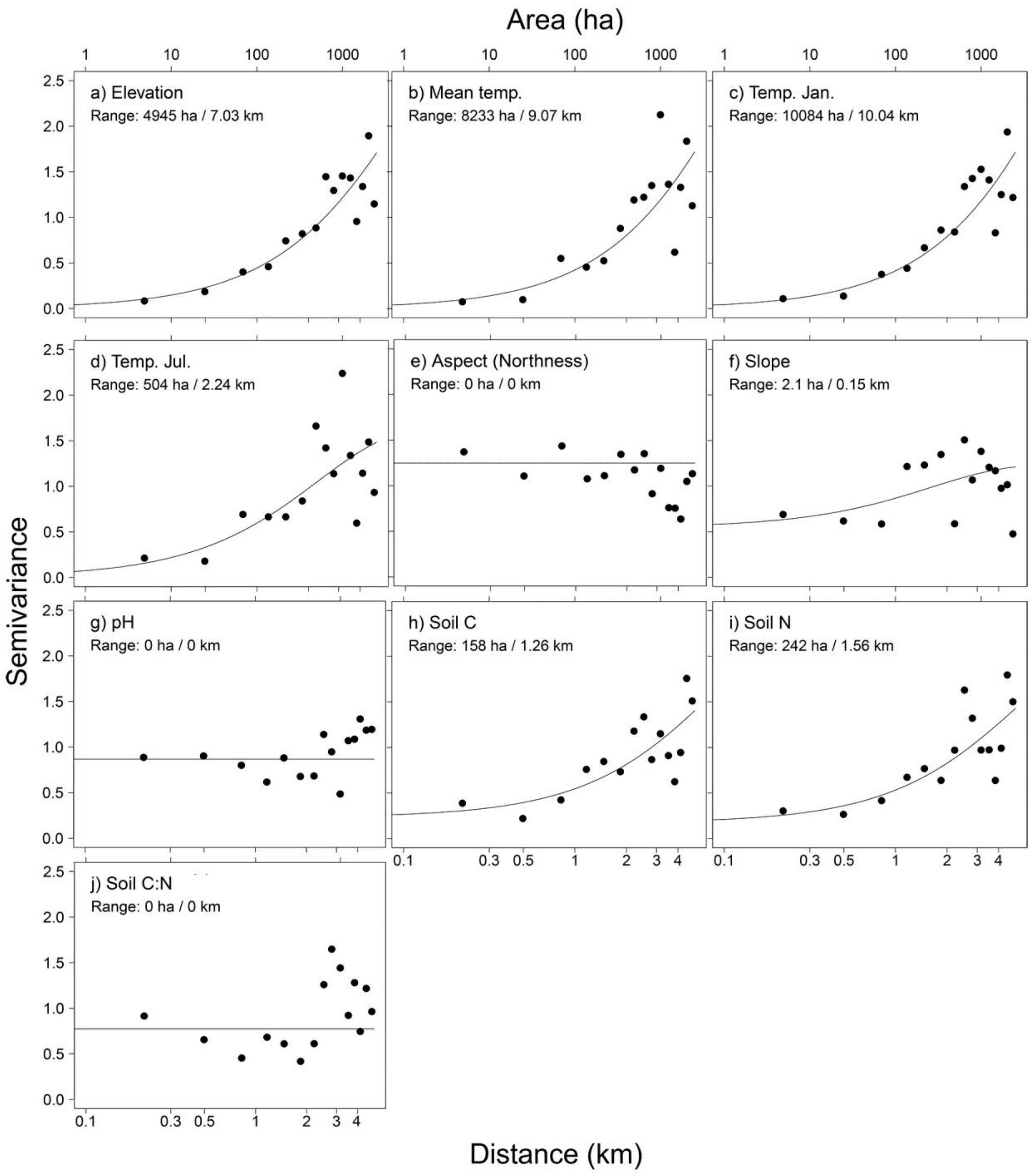

Supplementary Figure 4 | Semivariograms of environmental variables at the study site, showing the variability across spatial scales. Increasing semivariance values indicate spatial dependence, with the range giving the distance at which the semivariance levels off and reaches an asymptote. Variables such as elevation and temperature show strong spatial relationships up to scales of several thousand ha, indicating that they might be indicative of large-scale species turnover. Variables with small ranges such as slope, soil C and N might be indicative of turnover patterns that peak at smaller spatial scales. Variables with range values

of 0 such as aspect (northness), pH and soil C:N show a high variation at already at scales smaller than those considered in our study. *S*ample semivariances were calculated for all environmental variables up to a distance of 5 km, given that maximum distance between plots was 9 km, using the R package gstat. For comparability with the graphs on species-area relationship, variograms are on log(10) scale with corresponding midpoints of distance classes. The variograms were fitted with exponential models without nuggets at the untransformed distance scale except for models that resulted in singular models, which then were calculated with nugget-only models.

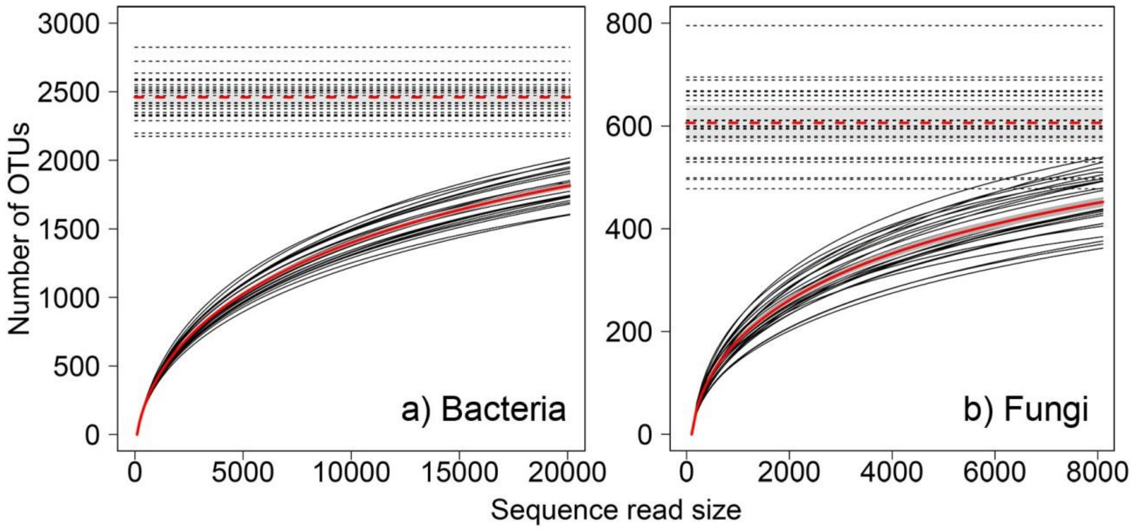

Supplementary Figure 5 | Rarefaction curves showing the number of OTUs sampled per plot based on the number of reads for a) bacteria and b) fungi. The dotted lines above the rarefaction curves indicate the number of OTUs expected to occur in each plot (Chao1). Red lines show average values across all plots (shaded areas indicate SE), showing that both taxa are equally well sampled within plots, with, on average, 74% (bacteria) and 75% (fungi) of the expected number of OTUs.

Supplementary Table 1 | Relative proportions (%) of the estimated overall number of species per taxon occurring in 1 and 10 ha of the GNNR

	1 ha		10 ha	
Taxon	All 27 plots	Reduced (17 plots)	All 27 plots	Reduced (17 plots)
Plants				
Woody plants	56	61 (+5)	85	88 (+3)
Herbaceous plants	40	45 (+5)	81	84 (+3)
Arthropods				
Araneae	30	33 (+3)	70	72 (+2)
Chilopoda	26	36 (+10)	64	78 (+14)
Diplopoda	35	27 (-8)	81	71 (-10)
Hymenoptera pred.	29	27 (-2)	76	88 (+12)
Hymenoptera parasit.	27	20 (-7)	64	55 (-9)
Formicidae	51	52 (+1)	81	82 (+1)
Cerambycidae	41	55 (+14)	80	90 (+10)
Curculioninae	14	31 (+17)	45	76 (+31)
Scolytinae	73	78 (+5)	93	95 (+2)
Lepidoptera	12	16 (+4)	46	57 (+11)
Fungi				
Archaerhizomycetes	83	82 (-1)	100	100 (0)
Ascomycota incertae sedis	67	70 (+3)	96	95 (-1)
Dothideomycetes	64	64 (0)	94	93 (-1)
Eurotiomycetes	63	60 (-3)	91	90 (-1)
Leotiomycetes	78	82 (+4)	100	100 (0)
Orbiliomycetes	75	84 (+9)	100	99 (-1)
Sordariomycetes	79	94 (+15)	100	100 (0)
Agaricomycetes	75	81 (+6)	100	100 (0)
Tremellomycetes	54	46 (-8)	89	84 (-5)
Wallemiomycetes	79	36 (-43)	100	68 (-32)
Glomeromycetes	63	77 (+14)	94	100 (+6)
Zygomycota	74	79 (+5)	97	97 (0)
Bacteria				
Acidobacteria	94	97 (+3)	99	100 (+1)
Actinobacteria	91	96 (+5)	99	100 (+1)
Alphaproteobacteria	92	96 (+4)	99	100 (+1)
Armatimonadetes	95	89 (-6)	100	99 (-1)
Bacteroidetes	95	88 (-7)	100	99 (-1)
Betaproteobacteria	92	92 (0)	99	99 (0)
Chlorobi	88	93 (+5)	100	100 (0)
Chloroflexi	87	87 (0)	98	98 (0)
Cyanobacteria	93	92 (-1)	100	99 (-1)
Deltaproteobacteria	95	94 (-1)	100	100 (0)
Elusimicrobia	90	90 (0)	99	99 (0)
Firmicules	93	93 (0)	100	100 (0)
Gammaproteobacteria	93	83 (-10)	100	98 (-2)

Gemmatimonadetes	92	92 (0)	100	99 (-1)
Planctomycetes	95	88 (-7)	100	99 (-1)
TM6	93	94 (+1)	100	100 (0)
Verrucomicrobia	90	94 (+4)	100	100 (0)
WCHB1-60	96	95 (-1)	100	100 (0)
WD272	94	96 (+2)	100	100 (0)
Plants (mean)	48	53 (+5)	83	86 (+3)
Arthropods (mean)	34	38 (+4)	70	76 (+6)
Fungi (mean)	71	71 (0)	97	94 (-3)
Bacteria (mean)	93	92 (-1)	100	99 (-1)

Data are based on the full dataset of all 27 study plots (average distance between plots 3.4 km) and a reduced dataset of the central 17 plots (average distance between plots 1.8 km). Values in parentheses indicate the difference between the relative proportions of the full and reduced datasets.

Supplementary Table 2 | Variation in topography, microclimate, and soil chemistry across the 27 study plots

Variable	Min.	Max.	Mean	SD
Elevation (m)	251	903	547	168
Mean annual temperature (°C)	15.1	18.0	17.0	0.8
Mean temp. January (°C)	0.6	4.5	2.7	1.0
Mean temp. July (°C)	23.5	26.9	25.8	0.8
Aspect (°)	7	358	185	103
Slope (°)	13	47	34	8
Soil pH	3.4	4.5	3.9	0.3
Soil C concentration (%)	1.8	9.9	3.4	1.8
Soil N concentration (%)	0.1	0.5	0.2	0.1
Soil C-N ratio	13.6	21.6	18	1.8

Full details of each study plot's characteristics are provided in Supplementary Data 1. For details on measurements, see ref.[1-2] in Supplementary References.

Supplementary References

1 Bruelheide, H. *et al.* Community assembly during secondary forest succession in a Chinese subtropical forest. *Ecol. Monogr.* **81**, 25-41 (2011)

2 Staab, M., Schuldt, A., Assmann, T., Bruelheide, H. & Klein, A.-M. Ant community structure during forest succession in a subtropical forest in South-East China. *Acta Oecol.* **61**, 32-40 (2014).
